# PD1 expression on bone marrow T‐cells in newly diagnosed Egyptian AML patients: Correlation with hematological parameters, aberrant antigens expression, and response to induction therapy

**DOI:** 10.1002/jha2.10

**Published:** 2020-05-26

**Authors:** Noha Bassiouny, Nour El‐Hoda, Ibtesam M Khalifa, Sara Ibrahim, Lamyaa Salem, Layla Annaka

**Affiliations:** ^1^ Department of Clinical Pathology Faculty of Medicine Ain Shams University Cairo Egypt; ^2^ Department of Internal Medicine and Clinical Hematology Faculty of Medicine Ain Shams University Cairo Egypt

**Keywords:** acute myeloid leukemia (AML), bone marrow (BM), complete remission (CR), flow cytometry, programmed cell death protein 1 (PD‐1)

## Abstract

**Background:**

Programed cell death protein 1 (PD‐1) is a key mediator for the development of T cell exhaustion that develops in response to persistent antigen stimulation.

**Aim:**

In this study, we measured PD1 expression on CD3 positive bone marrow T‐lymphocytes in newly diagnosis AML patients and its relation to clinical/ prognostic outcomes in addition to response to induction therapy (day 28).

**Methods:**

This study was conducted on 59 newly diagnosed AML patients and 20 healthy controls. Complete blood counts, flow cytometry using acute leukemia panel in addition to PD1 monoclonal antibodies were performed on bone marrow lymphocytes (CD3+), whereas cytogenetic/molecular studies were used to determine risk group. The patients’ remission status following induction therapy was determined.

**Results:**

PD1 was brightly expressed in 91.5% of the cases than control sample with highly significant difference (*P* = .001). A cutoff of 3.5 for mean fluorescence intensity was used to divide patients into two groups (higher vs normal PD1 expression). A significant difference between the two groups regarding platelet count and aberrant CD7 expression (*P* = .007 and .023, respectively) was found. Those normally expressed PD1 respond better to induction therapy.

**Conclusion:**

PD1 expression on BM T‐cells had a predictive value and providing an immunotherapeutic target for AML.

## 1 INTRODUCTION

Several malignant tumors are highly refractory to conventional therapies. The survival of tumors in several cases is assisted by checkpoint immunomodulation to maintain the imbalance between immune surveillance and cancer cell proliferation [1]. That is because tumors can adapt to immune pressure through the loss of antigenicity and immunogenicity as well as through their ability to create an immunosuppressive microenvironment. Therefore, distinct therapeutic strategies, depending on the mechanism of immune evasion by cancer cells, may be required for restoring productive cancer immunosurveillance [2].

Acute myeloid leukemia (AML) is a devastating blood cancer with 5‐year survival of only 25%. Targeting inhibitory mechanisms to unleash the patient's own antitumor immune response has achieved major success [3]. The two best known inhibitory immune checkpoints are cytotoxic T‐lymphocyte antigen‐4 (CTLA‐4) and the programmed cell death protein 1 receptor (PD‐1) [4]. Blockers of these checkpoints are rapidly becoming a highly promising cancer therapeutic approach that yields remarkable antitumor responses with limited side effects [1].

Physiologically, the PD‐1/PD‐L1 pathway has emerged as a result of the need to control the degree of inflammation at locations expressing the antigen, in order to secure normal tissue from damage. There is a remarkable expression of the PD‐1 protein on the surface of all activated T cells. When a T cell recognizes the antigen expressed by the MHC complex on the target cell, inflammatory cytokines are produced, initiating the inflammatory process. These cytokines result in PD‐L1 expression in the tissue, activating the PD‐1 protein on T cells leading to immune tolerance, a phenomenon where the immune system loses the control to mount an inflammatory response, even in the presence of actionable antigens [5].

Although this pathway is essential for maintaining peripheral T cell tolerance and is critical for attenuating autoimmunity and maintaining T cell homeostasis, it is also a deterrent to antitumor immunity. Advanced cancer patients who have failed all other therapies have impressive responses when treated with monoclonal antibodies (mAbs) that block this pathway, either as monotherapy or in combination with other mAbs that block signaling through CTLA‐4. PD1 (pembrolizumab and Nivolumab) or PDL1 (Durvalumab) inhibition therapies alone had limited clinical activity in AML and the hypomethylating agent (Azacitidine(AZA)) can promote immune recognition of tumor cells and increase expression of immune checkpoint molecules (PD1/PDL1) mediating resistance to AZA. It was found that combination of PD1/PDL1 inhibition therapies and azacitidine enhance antitumor activity and improve clinical outcome in relapsed/refractory AML and in newly diagnosed older AML patients [6, 7]. Immune checkpoint inhibition therapy can lead to activation of autoreactive T‐cells resulting in unique immune‐related adverse events, so combining MBG453; anti‐T‐cell immunoglobulin domain and mucin domain 3(anti‐TIM3) with hypomethylating agents was extremely well tolerated with no high grade immune‐related toxicities [8].

In this study, we measured PD‐1 expression on CD3 positive bone marrow (BM) T‐lymphocytes in newly diagnosis adult Egyptian AML patients and its relation to clinical/ prognostic parameters in addition to response to induction therapy (day 28).

## 2 MATERIALS AND METHODS

This study was conducted on 79 subjects (59 newly diagnosed adult AML patients and 20 age‐ and sex‐matched healthy controls), with their ages ranging from 18 to 77 years. AML was diagnosed by BM aspirate and biopsy using morphologic, cytochemical, immunophenotypic, and cytogenetic/molecular analyses. Diagnosis was made according to World Health Organization criteria 2016. After diagnosis; all included patients received the same induction chemotherapy protocol (induction therapy with Standard “7 + 3” regimens). This regimen combines a 7‐day continuous intravenous (IV) infusion of cytarabine (200 mg/m^2^ per day) with a bolus of an anthracycline given on days 1 through 3 (doxorubicin “Adriamycin” 60 mg/m^2^ for 3 days). New BM aspiration was done for all patients at day 28 to assess response to induction chemotherapy. Remission (responders group) was diagnosed by day 28 BM aspiration showing less than 5% blasts. All procedures performed in our study were in accordance with the ethical standards of the institutional and national research committee and with the 1964 Helsinki declaration and its later amendments or comparable ethical standards. Informed consent was obtained from all individuals who participated in the study. All patients were subjected to the following.

### 2.1 BM sampling at diagnosis

About 4‐5 mL BM aspirate was obtained and divided into:

- 0.5‐1 mL for Leishman smears
- 1.5 mL on K2‐EDTA for immunophenotyping
- 3 mL on Li‐Heparin for cytogenetic and molecular studies

### 2.2 Multiparameter flow cytometric analysis

**FIGURE 1:**
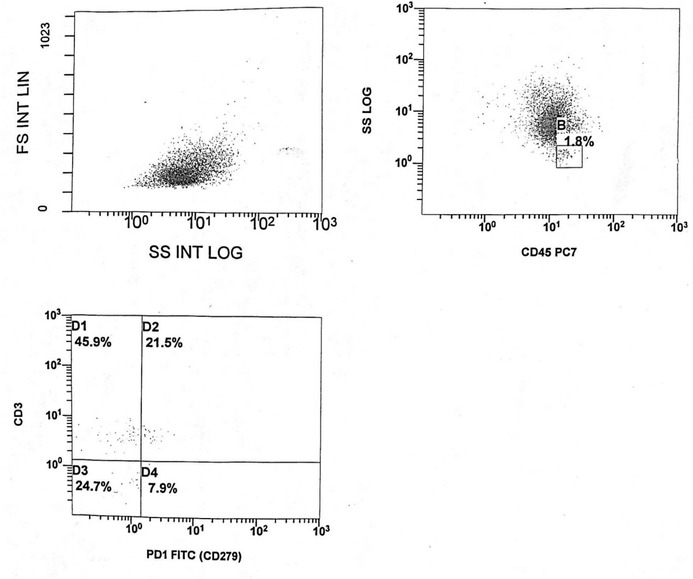
Bone marrow lymphocytes were gated using side scatter/CD45 expression (gate B). Those gated lymphocytes were analyzed for the percentage of CD3+ and expression of PD1 (in this case, BM lymphocytes were 1.8%, of which 21.8% double expressed CD3+PD1+ with MFI 2.8)

By NAVIOS 2 laser 6 colors flow cytometry (Beckman coulter, USA). The acute leukemia panel of fluorescein isothiocyanate (FITC)/phycoerythrin (PE) conjugated monoclonal antibodies (Beckman Coulter, life science, Hielach, USA); IgG1PE, CD45FITC, CD117PE, CD10FITC, CD19PE, HLA‐DR FITC, CD13PE, CD33PE, CD3FITC, CD7PE, C.TDTFITC, C.MPOFITC, CD14PE, CD15FITC, CD34PE, CD61 FITC, GLYCOPHORINPE, and CD56 PE (clone N901) were used for diagnosis and sub‐classification of AML. Schiff's reagent (Merck), erythrocyte lyses solution (FACS lyse BD‐Bioscience), perforating reagent tween 20, and phosphate buffer solution were used to prepare the solutions necessary to carry out the technique [9]. Gating was done on the residual normal BM lymphocyte population based on forward and side scatters and their bright expression of CD45. Those gated lymphocytes were analyzed for the percentages of CD3 + (PC5) and the expression of PD1 (VioBright FITC, clone 1.3.1.3) on these CD3+ lymphocytes (Figure 1).

### 2.3 Cytogenetic (FISH) analysis

At least 100 interphase nuclei were scanned for the detection of the signals by CytoVision automated cytogenetics platform (Leica Biosystems Richmond, USA). The used probes[Vysis] were RUNX1/RUNX1T1 (double fusion probe), PML/RARA, CBFB break apart probe, LSI MLL break apartand LSI BCR/ABL( single fusion probe). A cutoff value for diagnosis of positive results was >10% for single fusion probe and >3% for double fusion ones.

### 2.4 FLT3‐ITD/NPM1/C‐Kit mutational analysis

Genomic DNA was extracted using salting out method. PCR products with altered gel mobility were sequenced. All PCR products were sequenced with Rotor Gene PCR Analyzer (Qiagen, Germany) using the BIG DYE terminator V1.1 cycle sequencing kit.

### 2.5 Statistical analysis

In addition to descriptive analysis, data were analyzed using SPSS version 20 (International Business Machines Corporation, New York, 2010) statistical package,for analytical statistics; Man‐Whitney test, Kruskal‐Wallis test, Fisher's exact test, and correlation analysis (using spearman's method) were used in addition to receiver operating characteristic (ROC).

## 3 RESULTS

### 3.1 Laboratory and clinical data of the studied patients

The patients were classified according to the French‐American‐British (FAB) Cooperative Group Criteria and WHO cytogenetic/molecular classification 2016. According to cytogenetic risk group classification, 18 (30.5%) patients fell in the favorable risk group, 13 (22%) patients fell in the intermediate risk group, and 28 (47.5%) patients fell in the adverse risk group. Regarding their response to induction therapy at day 28, patients were divided into two groups: 40 (67.8%) patients achieved complete remission, whereas 19 (32.2%) patients were nonresponders (Table 1).

**TABLE 1:** Clinical and biological characteristics of the studied AML patients

Parameter		Range (Mean ± SD)
Age (years)		18‐77 (41.78 ± 15.5)
Clinical parameter		Number (percentage)
Sex	Male	31 (52.5%)
	Female	28 (47.5)
FAB subtypes	M0	5 (8.5%)
	M1	6 (10.2%)
	M2	33 (37.3%)
	M3	6 (10.2%)
	M4	4 (6.8%)
	M5	4 (6.8%)
	M7	1 (1.7%)
Cytogenetic risk group	Favorable	18 (30.5%)
	Intermediate	13 (22%)
	Adverse	28 (47.5%)
Response to induction therapy	Responder	40 (67.8%)
	Nonresponder	19 (32.2%)
Hematological parameters		Range [(Mean ± SD) or (Median IQR)]
	Hb (g/dL)	3.5‐12.3 (7.02 ± 1.81)
	TLC (× 10^3^/uL)	24 (3‐90)
	PLT (× 10^3^/uL)	4‐248 (63.66 ± 55.79)
	PB blast (%)	0‐95 (40.05 ± 27.61)
	BM blast (%)	16‐98 (63.92 ± 24.42)
Flow cytometric markers		Number (percentage)
PD1 expression on CD3+ cells MFI cutoff (3.5)	Positive	54 (91.5%)
	Negative	5 (8.5%)
HLA‐DR on myeloblasts	Positive	36 (61%)
	Negative	23 (39%)
Aberrant CD56 expression on myeloblasts	Positive	18 (30.5%)
	Negative	41 (69.5%)
Aberrant CD7 expression on myeloblasts	Positive	26 (44.1%)
	Negative	33 (55.9%)

Abbreviations: BM, bone marrow; FAB, French American British; Hb, hemoglobin; IQR, interquartile range; MFI, mean fluorescent intensity; PB, peripheral blood; PLT, platelets; SD, standard deviation; TLC, total leukocytic count.

**FIGURE 2:**
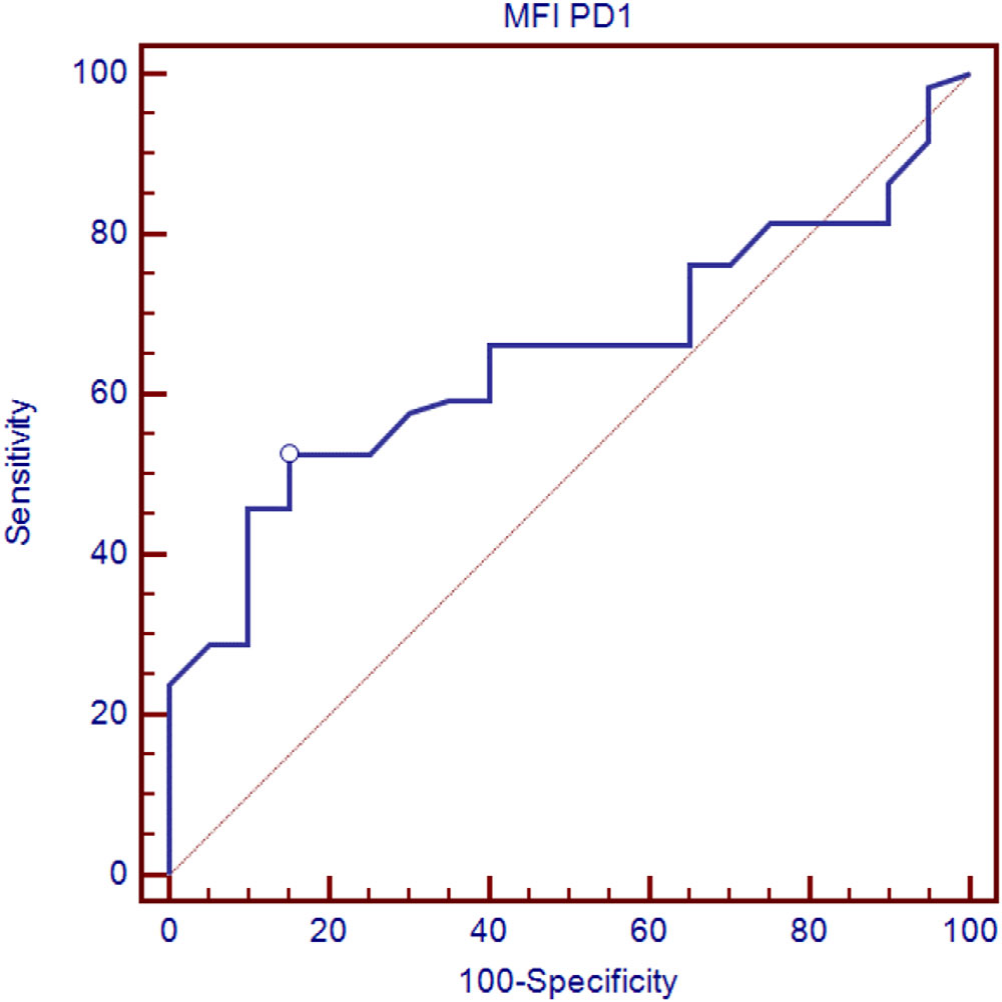
ROC curve of MFI PD1 expression on T‐lymphocytes

Median (range) of total leukocytic count (TLC), hemoglobin (Hb), platelet (PLT), and peripheral blood/BM blast cells percentage is listed in Table 1. HLA‐DR was expressed on myeloblasts of 36 (61%) patients. Aberrant expression of CD56 and CD7 on myeloblasts was noticed in 18 (30.5%) and 26 (44.1%) patients, respectively. Fifty‐four (91.5%) patients expressed PD‐1 on their CD3+ T‐lymphocytes with mean fluorescence intensity (MFI) of 4.06 ± 2.29. This expression was bright compared to the dim expression of the control samples (2.76 ± 1.04) with highly significant difference (*P* = .001). A Receiver operating characteristic (ROC) curve was constructed to discriminate patient and control samples regarding MFI expression of PD1 on CD3+ T‐cells. A cutoff of 3.5 was used to discriminate between both control and studied patient samples with 52.5% sensitivity and 85% specificity (Table 2; Figure 2).

**TABLE 2:** ROC curve of MFI of PD‐1 expression difference between AML patient and control BM samples

Area under the curve (AUC)	95% Confidence interval	*P*‐value	Significant difference	Cutoff	Sensitivity	Specificity
0.648	0.533 to 0.752	.0179	S	>3.5	52.54	85

### 3.2 PD1 expression in relation to different clinical/Laboratory parameters and response to induction therapy

Based on the PD‐1 expression (MFI) on BM CD3+ T lymphocytes (Table 3), patients were divided into two groups: a group with high PD‐1 expression (MFI >3.5%) and the other with normal PD‐1 expression (MFI <3.5%) compared to the control group. A statistical significance (*P* = .007) was found between the two groups regarding platelet count, with lower platelet count in those with higher PD1 expression. A significant difference (*P* = .023) was noticed between the two groups concerning aberrant CD7 expression on myeloblasts of the high PD‐1 expression group; however, no statistical significance (*P* = .964) was found regarding HLA‐DR expression on myeloblasts. A significant difference (*P* = .04) between different cytogenetic risk groups and PD‐1 expression was found, where favorable risk group showed lower PD‐1 expression than that of the intermediate and adverse risk groups. Regarding response to induction therapy (day 28), patients with normal PD‐1 expression responded better to induction therapy (ie, complete remission) than patients with high PD‐1 expression with a significant difference (*P* = .025).

**TABLE 3:** Clinical, hematological parameters, flow cytometric markers, cytogenetic risk, and response to induction therapy according to BM T‐lymphocytes PD1 expression

		PD1 expression (MFI)			
Parameters		Normal PD‐1 expression (MFI <3.5) n = 28	High PD‐1 expression (MFI >3.5) n = 31	*P*‐ value	Significant difference
Sex					
Females		14 (50.0%)	14 (45.2%)	.710	NS
Males		14 (50.0%)	17 (54.8%)		
Age					
<60 Years		20 (71.4%)	25 (80.6%)	.406	NS
>60 years		8 (28.6%)	6 (19.4%)		
Hematological parameters					
Hb (g/dL) mean ± SD		7.1 ± 1.9	6.9 ± 1.7	.661	NS
TLC(× 10^3^/uL) Median (IQR)		29.5 (4.8‐92)	12.6 (2.3‐90)	.404	NS
PLT(× 10^3^/uL) Median (IQR)		65.5 (46.5‐126)	27 (18‐60)	.007	S
PB blast % mean ± SD		32.5 (62‐32.5)	42 (64‐42)	.703	NS
BM blast % mean ± SD		72.5 (82.5‐72.5)	68 (85‐68)	.909	NS
FAB number (%)	0 1 2 3 4 5 7	2 (7.1%) 5 (17.9%) 15 (53.5%) 1 (3.6%) 2 (7.1%) 2 (7.2%) 1 (3.6%)	3 (9.7%) 1 (3.2%) 18 (58.1%) 5 (16.1%) 2 (6.5%) 2 (6.4%) 0 (0.0%)	.381 ^F^	NS
Flow cytometric markers (Number of cases with positive CD marker (%)					
HLA‐DR expression		17(60.7%)	19 (61.3%)	.964	NS
Aberrant CD7 expression		8 (28.6%)	18 (58.1%)	.023	S
Aberrant CD56 expression		8 (28.6%)	10 (32.3%)	.759	NS
Cytogenetic risk groups (number %)					
Favorable		12 (42.8%)	6 (19.3%)	.04	S
Intermediate		4 (14.2%)	9 (29%)		
Adverse		9 (32.1%)	19 (61.2%)		
Response to induction therapy (day 28) (number %)					
Nonresponder		5 (17.9%)	14 (45.2%)	.025	S
Responder		23 (82.1%)	17 (54.8%)		

Abbreviations: BM, bone marrow; F, Fisher exact test; FAB, French American British; Hb, hemoglobin; IQR, interquartile range; MFI, mean fluorescent intensity; PB, peripheral blood; PLT, platelets; S, significant; SD, standard deviation; TLC, total leukocytic count; NS, nonsignificant.

## 4 DISCUSSION

Novel immunotherapeutic strategies are rapidly evolving for treatment of the devastating acute myeloid leukemia. T‐cell dysfunction has been noticed in various hematological neoplasm and has been put into the context of T‐cell exhaustion as a result of increase expression of several inhibitory receptors in combination with defect effector function and finally apoptosis [10]. The PD‐1 is a key mediator for such T‐cell exhaustion that develops in response to persistent antigen stimulation, including cancer [3]. In this study, we measured PD‐1 expression on CD3 positive BM T‐lymphocytes in newly diagnosed adult Egyptian AML patients and its relation to clinical/prognostic parameters in addition to response to induction therapy (day 28).

Limited studies were carried on the immune response in BM of AML patients; although in a majority of patients, AML is derived from myeloid hematopoietic progenitors and rapidly grows in BM before mobilizing to peripheral blood; therefore understanding the anti‐leukemia immune response within the BM of AML patients is likely to be a key to develop immune therapeutics for leukemia, and therefore in this study, we investigated the inhibitory checkpoint (PD‐1) T cell within BM of AML patients [11].

In our study, examination of CD3 positive T‐cells was done without differentiation in reference to the study done by Lion et al [12] who found that T cells of newly diagnosed AML patients were not significantly different from those of age‐matched healthy controls, suggesting that increased T‐cell differentiation could be induced by a long standing contact with AML cells (i.e, chronic stimulation). Inhibitory molecules (e.g, PD1) on T‐cells have not been studied as broadly as on CD8 T‐cells; however, the same molecules were also seen to play a role in CD4 T‐cells as to CD8 cells. Persistent antigen exposure can induce a dysfunction state in CD4 T‐cells that correlate with PD1 expression; it was found that Treg were unlikely to account for the observed increasein PD1 expression on CD4 T‐cells, as isolated Treg from healthy controls has been reported to express PD1 intracellular [13]. The increase in inhibitory PD1 molecules was a surrogate for a shift toward different effector T‐cells instead of a significant T‐cell exhaustion; therefore such exhausted T‐cells might be as a result of chronic activation [14].

In this study, on evaluating PD‐1 expression on CD3+ BM T‐lymphocytes between patients and control samples, patients’ samples showed bright expression compared to the dim expression of the control samples. A cutoff of MFI 3.5 was used to discriminate between both control and studied patient samples. According to the PD‐1 expression (MFI) on BM CD3+ T lymphocytes of AML patients, there were two groups: a group with high PD‐1 expression (MFI >3.5%) and the other with normal PD‐1 expression (MFI <3.5%).

Patients with normal PD‐1 expression responded better to induction therapy (ie, complete remission) than patients with high PD‐1 expression with a significant difference. This could be explained by a study done by Tan et al [15] who found a decreased tendency of exhausted T cells (PD1+CD244+, PD1+CD57+) in AML‐complete remission group and found a particular influence on CD8+ exhausted T‐cells, suggesting a poor anti‐leukemia immune response in these patients; however, in our study we did not measure expression of PD1 or exhaustion markers on BM lymphocytes at day 28 for further evaluations.

Regarding platelet count that was significantly lower in those with higher PD‐1 expression, a study done by Elif et al [16] who measured serum PD‐1 and PD‐L1 in patients with idiopathic thrombocytopenic purpura (ITP) revealed that there was a positive correlation between serum PD‐1 levels and platelet count, which needs further studies to correlate serum PD1 and platelet count in AML patients. In this study, aberrant CD7 expression on myeloblasts was found in those with higher PD‐1 expression on their T‐cells; an early study described an increase of some activation markers (HLA‐DR, CD69, CD71, and CD57) on T‐cells at diagnosis [17]. This was in line with data from gene expression profiling of T‐cells providing some hints at aberrant T‐cell markers in AML patients [18]. Whether aberrant T‐cell markers on myeloblasts of AML patients are associated along with PD L1 and PD1 on T‐cells is a question for further larger cohort studies.

## 5 CONCLUSION

This study determined a cutoff value of MFI 3.5 to differentiate PD‐1 expression level on CD3+ BM T‐lymphocytes of newly diagnosed adult Egyptian AML patients. Higher PD‐1 expression was associated with lower platelet count, aberrant CD7 expression on their myeloblasts, and poor response to induction therapy.

## CONFLICT OF INTEREST

The authors declare no conflict of interest.
